# Marked enhancement of lysosomal targeting and efficacy of ErbB2-targeted drug delivery by HSP90 inhibition

**DOI:** 10.18632/oncotarget.7231

**Published:** 2016-02-07

**Authors:** Srikumar M. Raja, Swapnil S. Desale, Bhopal Mohapatra, Haitao Luan, Kruti Soni, Jinjin Zhang, Matthew A. Storck, Dan Feng, Timothy A. Bielecki, Vimla Band, Samuel M. Cohen, Tatiana K. Bronich, Hamid Band

**Affiliations:** ^1^ Eppley Institute for Research in Cancer and Allied Diseases, Omaha, Nebraska, USA; ^2^ Department of Pharmaceutical Sciences and Center for Drug Delivery and Nanomedicine, Omaha, Nebraska, USA; ^3^ Department of Genetics, Cell Biology and Anatomy, College of Medicine, University of Nebraska Medical Center, Omaha, Nebraska, USA; ^4^ Departments of Biochemistry and Molecular Biology, Pathology and Microbiology and Pharmacology and Neuroscience, College of Medicine, Omaha, Nebraska, USA; ^5^ Department of Pathology and Microbiology, University of Nebraska Medical Center, Omaha, Nebraska, USA; ^6^ Robert H. Lurie Comprehensive Cancer Center, Northwestern University, Chicago, Illinois, USA

**Keywords:** breast cancer, ErbB2, targeted drug delivery, HSP90, Trastuzumab

## Abstract

Targeted delivery of anticancer drugs to tumor cells using monoclonal antibodies against oncogenic cell surface receptors is an emerging therapeutic strategy. These strategies include drugs directly conjugated to monoclonal antibodies through chemical linkers (Antibody-Drug Conjugates, ADCs) or those encapsulated within nanoparticles that in turn are conjugated to targeting antibodies (Antibody-Nanoparticle Conjugates, ANPs). The recent FDA approval of the ADC Trastuzumab-TDM1 (Kadcyla®; Genentech; San Francisco) for the treatment of ErbB2-overexpressing metastatic breast cancer patients has validated the strong potential of these strategies. Even though the activity of ANPs and ADCs is dependent on lysosomal traffic, the roles of the endocytic route traversed by the targeted receptor and of cancer cell-specific alterations in receptor dynamics on the efficiency of drug delivery have not been considered in these new targeted therapies. For example, constitutive association with the molecular chaperone HSP90 is thought to either retard ErbB2 endocytosis or to promote its recycling, traits undesirable for targeted therapy with ANPs and ADCs. HSP90 inhibitors are known to promote ErbB2 ubiquitination, targeting to lysosome and degradation. We therefore hypothesized that ErbB2-targeted drug delivery using Trastuzumab-conjugated nanoparticles could be significantly improved by HSP90 inhibitor-promoted lysosomal traffic of ErbB2. Studies reported here validate this hypothesis and demonstrate, both *in vitro* and *in vivo*, that HSP90 inhibition facilitates the intracellular delivery of Trastuzumab-conjugated ANPs carrying a model chemotherapeutic agent, Doxorubicin, specifically into ErbB2-overexpressing breast cancer cells, resulting in improved antitumor activity. These novel findings highlight the need to consider oncogene-specific alterations in receptor traffic in the design of targeted drug delivery strategies. We suggest that combination of agents that enhance receptor endocytosis and lysosomal routing can provide a novel strategy to significantly improve therapy with ANPs and ADCs.

## INTRODUCTION

Selective overexpression of certain plasma membrane receptors on cancer cells presents opportunities for targeted delivery of cytotoxic agents that are either directly linked to targeting ligands/antibodies or are encapsulated within nanometer-sized drug delivery vehicles decorated with targeting ligands/antibodies [1]. Receptor tyrosine kinases such as EGFR and ErbB2 are overexpressed in many cancers and are being increasingly targeted with targeted drug delivery modalities, including Antibody-Drug-Conjugates (ADCs) and nano-particulate drug delivery systems. Trastuzumab-DM1 (Kadcyla^®^, Genentech, San Francisco) is an example of an ADC developed against ErbB2-overexpressing cancers in which a cytotoxic drug (Emtansine) is chemically conjugated to humanized anti-ErbB2 monoclonal antibody Trastuzumab (Genentech) [2]. Success of drug delivery using ADCs or ANPs is predictably dependent on robust endocytosis of the receptor-bound drug and trafficking from early endosomes to lysosomes, where drug conjugate undergoes cleavage or nanoparticles optimally release their cytotoxic drug cargo, followed by drug entry into the cytosol [3-5]. In normal cells, endocytosis and receptor trafficking are highly regulated processes mediated and regulated by a plethora of cellular factors. Receptors are endocytosed *via* clathrin-dependent or non clathrin-dependent pathways [6, 7]. Following endocytosis, receptors are either routed to the lysosomes or recycled back to the cell-surface, processes regulated among other factors by receptor ubiquitination by E3 ubiquitin ligases (such as Cbl) or de-ubiquitination by AMSH or USP8 [8-13]. Endosomal Sorting Complex Required for Transport (ESCRT) proteins recognize ubiquitin-tagged receptor cargo for sorting into inner vesicles of the multivesicular body for eventual transport to lysosomes [9, 10, 14, 15]. Various Rab-family GTPases, functioning at distinct vesicular trafficking steps, also play critical roles in directing the traffic of endocytosed cargo towards recycling *vs*. lysosomal pathways [16, 17]. Cancer cell-specific alterations (mutations, overexpression) in receptors themselves or in their traffic regulators can alter the balance between receptor recycling and lysosomal trafficking [8, 18]. While the focus of studies to optimize ANP and ADC design has been on the design of receptor-targeted drug delivery systems [19-22], the consequence of altered recycling *vs*. lysosomal trafficking behavior of targeted receptors, and consequently the efficiency of drug delivery, have received little attention. In this study, we illustrate the importance of receptor traffic on targeted drug delivery, using ErbB2-overexpressing breast tumor targeting through Trastuzumab as a model.

The oncogenic receptor tyrosine kinase ErbB2, overexpressed in over 20% of breast cancers, has served as a major target for the development of targeted drug delivery strategies [23-26]. ErbB2, however, is thought to be impaired in endocytosis and to rapidly recycle back to the cell surface [27-31], which can significantly dampen the efficacy of ErbB2-targeted drug delivery. Altered trafficking of ErbB2 is believed to be due in part to its constitutive association with HSP90 [31-36], which can be anticipated to dampen the efficacy of ErbB2-targeted drug delivery. We therefore hypothesized that HSP90 inhibition will enhance ErbB2-targeted drug delivery by promoting the endocytic uptake of ErbB2-bound nano-encapsulated cargo and facilitating its re-routing from a recycling pathway to the lysosomes. Using Trastuzumab-conjugated nanogels (Trast-NG) encapsulating the DNA-damaging drug Doxorubicin (DOX) as a model chemotherapeutic, we demonstrate through *in vitro* and *in vivo* studies that HSP90 inhibition can indeed lead to an enhancement of targeted delivery of DOX specifically into ErbB2-overexpressing breast cancer cells. As a consequence, a sub-therapeutic and non-toxic dose of the HSP90 inhibitor 17AAG markedly improves the efficacy of ErbB2-targetd nanogels *in vivo*.

## RESULTS

### Preparation and characterization of Trast-NG

**Figure 1:**
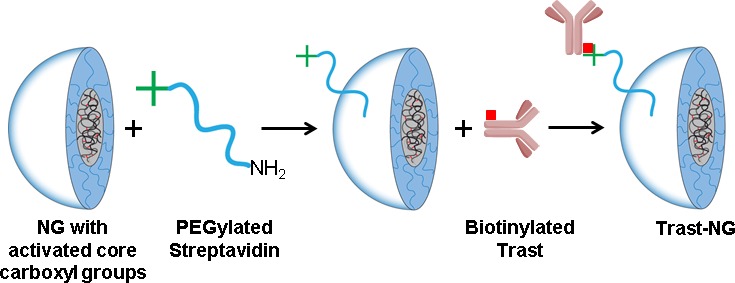
Scheme for the synthesis of Trast-NG conjugate *via* streptavidin-biotin complex

The core-shell NGs studied here were synthesized *via* self-assembly of doubly-hydrophilic poly(ethylene glycol)-*b*-poly(methacrylic acid) (PEG-*b*-PMA) block copolymers in the presence of a condensing agent (CaCl_2_), followed by chemical crosslinking of the poly-ion chains and removal of the condensing agent, as previously described [37, 38]. The resulting NGs averaged 110 nm in diameter and had a net negative charge (ζ-potential = −22 mV). The core of such (PEG-*b*-PMA)-based NGs comprises of a swollen network of cross-linked poly-ion chains bearing carboxylic groups and is surrounded by a shell of hydrophilic PEG chains (Figure 1). These NGs can incorporate very large amounts (∼30% by weight) of water-soluble drugs such as DOX through combination of electrostatic and Van der Waals interactions [38]. The NGs were functionalized with streptavidin by inserting an NH_2_-PEG-streptavidin linker. Since NGs shell formation requires ∼7.5 kDa PEG chains, we used longer chain PEG (M_w_ = 10 kDa) to reduce potential steric interference with receptor binding by conjugated antibodies. The average particle size of streptavidin-conjugated NGs was 137 ± 4 nm (polydispersity Index = 0.09, ζ-potential = −19.0 ± 2.3 mV), which is only slightly larger than that of precursor NGs. Biotinylated Trast or control IgG was then coupled with streptavidin-modified NGs. The final product was purified using FPLC; the chromatogram confirmed a very high yield with little residual free Trast or IgG (Figure S1). Purified Trast-NGs had an average particle size of 173 ± 3 nm (polydispersity Index = 0.08, ζ-potential = −20.2 ± 3.1 mV), whereas the control IgG-NGs had a size of 177 ± 2 nm (polydispersity Index = 0.07, ζ-potential = −19 ± 2.2 mV). After DOX loading, the average particle size of Trast-NGs decreased to 154 ± 4 nm (polydispersity Index - 0.19, ζ-potential = −4.1 ± 0.9 mV), whereas the particle size of IgG-NGs was 157 ± 8 (polydispersity Index = 0.24, ζ-potential = −2.7 ± 1.3 mV). The reduction in particle size and net negative charge of NGs after DOX loading are consistent with neutralization and condensation of PMA segments by DOX.

**Figure 2:**
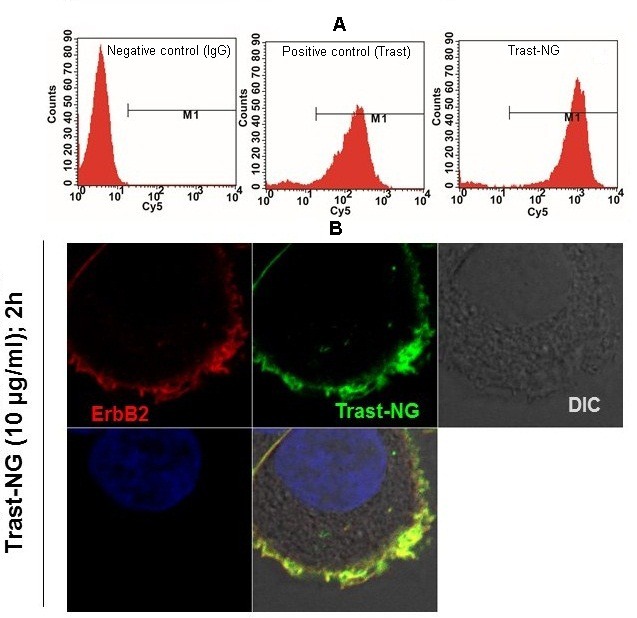
Trast-NG retains its ability to specifically bind to ErB2 **A.** ErbB2-overexpressing breast cancer cell line SKBr-3 was left untreated or treated with biotinylated Trast or Trast-NG on ice. The bound Trast or Trast-NG was detected using a Cy5-labeled anti-human secondary Ab alone. Samples were subjected to FACS analysis. Untreated cells stained with the Cy5-labeled secondary Ab served as a negative control, whereas biotinylated Trast served as a positive control. **B.** Binding of Trast-NG to ErbB2-overexpressing breast cancer cells was confirmed by confocal microscopy. ErbB2-was visualized using a mouse monoclonal antibody directed against the C-terminal antigenic region of ErbB2 (BD Pharmingen), followed by Alexa594-conjugated anti-mouse secondary antibody. The Trast-NG bound to the ErbB2 was detected using a FITC-conjugated goat anti-human secondary antibody to detect the Fc portion of the Trast-conjugated to the NG.

Conjugation of Trast to NG did not compromise its ability to specifically bind to ErbB2 receptors overexpressed on human breast adenocarcinoma SKBr-3 cells as confirmed by flow cytometry (FACS) (Figure 2A) and confocal immunofluorescence microscopy (Figure 2B and S2). In the latter analyses, two-color imaging showed complete colocalization of direct ErbB2 staining (stained in red) with that of bound Trast-NGs (stained in green) (Figure 2B and S2), demonstrating the ErbB2-specific binding of Trast-functionalized NGs.

To further explore the specificity of Trast-NGs, we compared the extent of growth inhibition (MTT dye incorporation) of ErbB2-overexpressing (BT-474) *vs*. ErbB2-low (MCF-7) breast cancer cell lines by DOX encapsulated within control IgG-NGs *vs*. Trast-NG (Table S1). The IC_50_ value of DOX-loaded Trast-NG (Trast-NG/DOX) against BT-474 cells was 6-fold lower compared to that of DOX-loaded NG (NG/DOX), while the two NGs inhibited MCF-7 cell growth with comparable IC_50_ values (Table S1). These experiments confirmed the ability of Trast-NG to deliver chemotherapeutic payloads to ErbB2-overexpressing breast cancer cells with high specificity.

### Kinetics of the endocytic uptake of Trast-NG into ErbB2-overexpressing breast cancer cells

**Figure 3:**
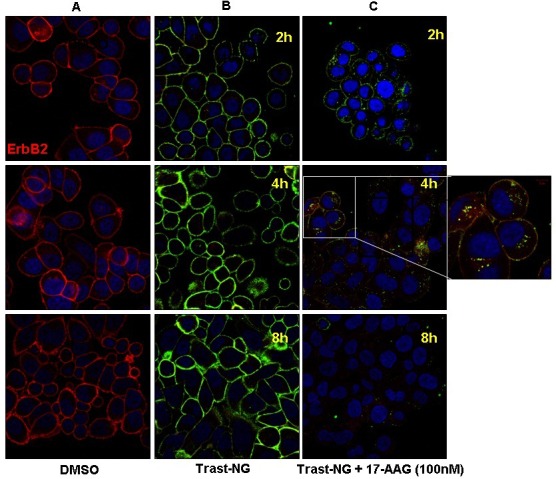
The HSP90 inhibitor 17AAG promotes the internalization and lysosomal degradation of Trast-NG ErbB2-overexpressing SKBr3 cells were plated on glass coverslips and incubated with Trast-NG without any encapsulated drug for 1h to allow binding to the cell surface. 17-AAG (100 nM) was then added for the indicated time points, after which the slides were washed and fixed, stained as described in the legend for Figure 2. The slides were analyzed by confocal immunofluorescence microscopy. The inset within panel C shows Trast-NG accumulating in punctate intracellular vesicles following 17-AAG treatment.

ErbB2 is thought to be impaired for endocytosis or to primarily recycle [27-31], whereas HSP90 inhibitors promote ErbB2 entry into lysosomes [31-36]. To assess the ability of Trast-NGs to access lysosomes by themselves or in the presence of HSP90 inhibitor 17AAG, we compared the kinetics of the endocytic uptake of Trast-NG into ErbB2-overexpressing cell line SKBr-3, using confocal immunofluorescence analysis. To avoid any impact of DOX, we used empty Trast-NGs for these analyses. Cells were either left untreated or treated with 10 μg/mL of free Trast or Trast-NG (based on protein concentration) for 2, 4 or 8 hours in absence or presence of 17-AAG (100 nM) followed by confocal imaging (Figure 3). In the absence of 17AAG, Trast-NG (stained in green) was found primarily at the cell surface throughout the 8h time course, with relatively little internalization, comparable to that seen with free Trast (Figure 3, second *vs*. first column). In contrast, cells incubated with Trast-NG in the presence of 17-AAG demonstrated rapid internalization of ErbB2 (stained in red) with notable internal staining at 2 h and nearly complete intracellular staining at 4h; as expected, ErbB2 levels decreased by 8h, reflecting the destabilization due to HSP90 inhibition [31, 32, 39] (Figure 3, last column). Loss of green Trast signal was concomitant with loss of ErbB2 (red signal), suggesting that, upon HSP90 inhibition, Trast-NGs follow the endocytic itinerary of ErbB2 receptor into lysosomes [31-36]. To assess if this is the case, we examined the colocalization of internalized Trast-NGs (stained in green) with the lysosomal marker LAMP-1 (stained in red) in SKBr-3 cells. Confocal Immunofluorescence analysis (Figure S3) confirmed that Trast-NGs indeed entered LAMP-1-positive compartments in the presence of 17AAG. These analyses demonstrate the strong positive impact of HSP90 inhibition to promote the internalization and lysosomal targeting of Trast-NG, a property desirable to facilitate the disintegration of NGs and release of their encapsulated drugs [3-5].

### HSP90 inhibitor-facilitated delivery of DOX into ErbB2-overexpressing breast cancer cells

Having verified our central hypothesis that HSP90 inhibition facilitates the routing of Trast-NGs (without drugs) into lysosomes, we evaluated the ability of 17-AAG to enhance the lysosomal delivery of NG-encapsulated DOX. The ErbB2-overexpressing 21MT-1 cells or the ErbB2-low MCF7 cells (specificity control) were incubated with increasing concentrations of Trast-NG/DOX for 6 h, after which the cells were either left alone (control) or treated with 100 nM 17-AAG for 18 h to trigger the internalization and lysosomal routing of targeted NG cargo. Biological effects were assessed by evaluating DOX-induced G2/M arrest using FACS.

**Figure 4:**
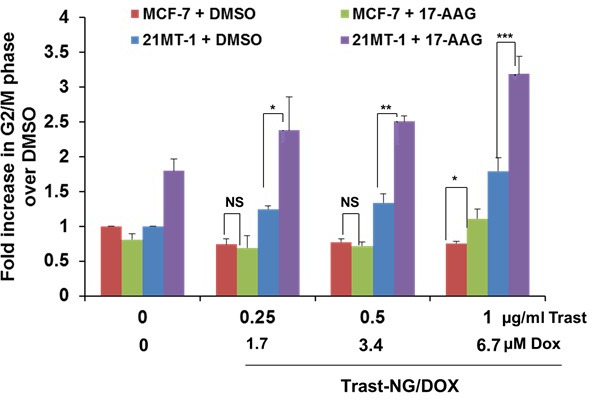
HSP90 inhibition potentiates the effect of Trast-NG/DOX on ErbB2-expressing breast cancer cells The ErbB2-overexpressing 21MT-1 or ErbB2-low MCF-7 cells were plated in 6-well plates and triplicate wells were treated with the indicated concentrations of Trast-NG/DOX for 6h. Unbound Trast-NG/DOX was washed out and cells were cultured further in DOX-free media without or with 17-AAG (100 nM) for 18 h followed by the incubation with nanogel-free media. Cell cycle analysis was performed after 48 h of treatment. Shown are the fold increase in the % of cells in G2/M phase of the cell cycle for each treatment condition over DMSO control (the color coding is indicated inside the histogram). The number of asterisks indicates increasing significance (*, *p* < 0.05; **, *p* < 0.005; ***, *p* < 0.0005); NS, not significant.

While Trast-NG/DOX treatment of 21-MT1 cells led to a dose-dependent increase in the percentage of cells arrested at the G2M phase, little effect was seen on MCF7 cells, especially at lower concentrations of Trast-NG/DOX (Figure 4). While inclusion of 17AAG had no impact on the effect of lower concentrations of Trast-NG/DOX on MCF7 cells, a statistically significant increase in G2M cells was noted in 21-MT1 cells treated with identical concentrations of Trast-NG/DOX (Figure 4). Thus, inclusion of 17AAG markedly increased the biological impact of Trast-NG/DOX, relatively selectively against the ErbB2-overexpressing cell line.

### Potentiation of the antitumor efficacy of Trast-NG/DOX by 17 AAG in an ErbB2-overexpressing breast cancer cell line xenograft model in mice

To examine if HSP90 inhibition potentiates the antitumor efficacy of Trast-NG/DOX *in vivo*, we treated mice bearing xenografts of ErbB2-overexpressing BT-474 or ErbB2-low MCF-7 breast cancer cell lines with Trast-NGs/DOX with or without 17AAG. In separate experiments (with 10 mice/group), we either measured changes in tumor volume, or also followed the treated animals for survival. Control treatments included: 1) 5 % dextrose (negative control); 2) Trast alone (positive control); 3) IgG-NG/DOX (non-targeted NG) and; 4) IgG-NG/DOX plus 17-AAG. A total of four treatments (intravenous injections) were given at 4-day intervals at a NG dose equivalent to 6 mg of DOX/kg (determined as the maximum tolerated dose for this treatment schedule) and 3 mg/kg Trast (where applicable). In the cohorts receiving 17-AAG, this drug was administered at 1 mg/kg 2 h after Trast-NG/DOX or IgG-NG/DOX injection.

**Figure 5:**
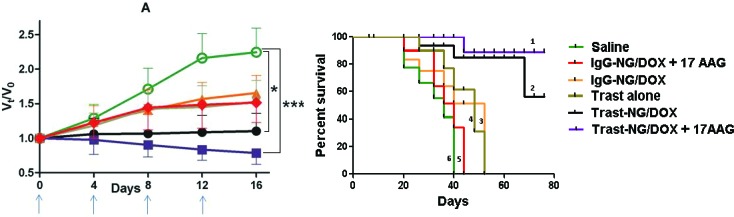
*In vivo* antitumor efficacy of Trast-NG/DOX and enhancement by sequential administration of 17-AAG against ErbB2-overexpressing breast cancer xenografts BT-474 xenografts were established by orthotopic injection of cells in mammary glands of female Nude mice, and the following treatments intravenously administered after tumors had grown between 200 and 300 mm3 in volume: Trast-NG/DOX + 17-AAG (■) or Trast-NG/DOX (•) or IgG-NG/DOX + 17-AAG (▼) or IgG-NG/DOX (▲) or Trast alone (♦) or Control. Drug formulations were injected in100 μl at a dose of 6 mg DOX or 3 mg Trast equivalents/kg body weight 4 times at 4-day intervals as indicated by the arrows. 17-AAG at a dose of 1mg/kg was given 2 h after NG formulation administration. Changes in tumor volume **A.** are presented as a fold-ratio compared baseline for each animal on day 0 of treatment. Values represent mean ± SEM. Kaplan-Meier analysis of the overall survival in Trast-NG/DOX + 17-AAG (1) or Trast-NG/DOX (2) or IgG-NG/DOX (3) or IgG-NG/DOX + 17-AAG (4) or Trast alone (5), 5 % dextrose control group (6) is shown **B.**. * or ** indicate a statistically significant difference between the indicated groups. *** indicates a statistically significant difference between Trast-NG/DOX and Trast alone.

The changes in the relative tumor volume for mice bearing BT-474 (ErbB2-high) tumors are shown in Figure 5A (data combines results from two independent experiments). To confirm the *in vivo* selectivity of the targeted NG to deliver DOX to ErbB2-overexpressing tumors, we also evaluated the effect of treatments with Trast-NG/DOX in comparison to the untargeted IgG-NG/DOX, on mice with MCF-7 (ErbB2-low) xenografts (Figure S4). Student's *t*-test was run on the tumor relative volume change data at day 16, comparing each treatment groups against 5% Dextrose control. Treatment of mice carrying BT-474 xenografts with Trast-NG/DOX alone led to a statistically significant (*p* < 0.05) tumor growth inhibition when compared to the control groups (*p*-value = 0.0159; data pooled from 2 independent experiments) (Figure 5A). Sequential Trast-NG/DOX followed by 17AAG treatment led to further enhancement of anti-tumor activity as compared to Trast-NG/DOX alone group and markedly increased statistical significance relative to control group (*p* = 0.0005). Remarkably, Trast-NG/DOX + 17AAG treatment led to an actual reduction in tumor volume (shrinkage) compared to the pre-treatment tumor volume, clearly observed at later time points (Figure 5A), although this difference did not reach statistical significance (*p* = 0.293).

Compared to results with BT-474 xenografts, mice bearing MCF-7 xenografts exhibited a small albeit statistically significant (*p* = 0.03 *vs*. control) reduction in tumor volume upon treatment with Trast-NG/DOX; however, the difference in tumor volume among groups treated with IgG-NG/DOX *vs*. Trast-NG/DOX groups was not significant (*p*-value = 0.397) (Figure S4). Comparative analysis of BT-474 *vs*. MCF-7 xenografts establishes the advantage of Trast-based targeting against an ErbB2+ tumor, and clearly demonstrates the enhancement of the Trast-NG/DOX efficacy by sequential post-treatment with a small dose (which by itself has no measurable effect on tumor growth; not shown) of the HSP90 inhibitor 17AAG.

Kaplan-Meier analysis of survival data at the end of the experiment (76 days; censoring date) clearly showed that BT-474 xenograft tumor-bearing mice treated with Trast-NG/DOX + 17-AAG had a substantially longer survival (> 88%) compared to the Trast-NG/DOX group (56.8%) (Figure 5B). None of the mice in other treatment groups (5 % dextrose, Trast alone, IgG-NG/DOX and IgG-NG/DOX + 17-AAG) survived until the censoring date (0% survival) (Figure 5B). The median survival (in days), the associated Confidence Intervals and the p-values are shown in Table S2. Overall, our data clearly shows the superiority of Trast-NG/DOX over untargeted NG/DOX and suggests that combined treatment with HSP90 inhibitors can vastly improve survival times.

**Figure 6:**
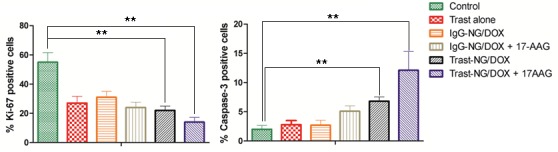
Administration of Trast-NG/DOX reduces the cell proliferation and promotes the apoptosis in BT-474 xenograft tumors and its activity is enhanced by 17AAG Post-treatment tumor sections were stained for Ki-67 and caspase-3 and % Ki-67-positive **A.** and % caspase-3-positive **B.** cells were calculated based on enumeration of at least 1000 cells. Tumors from three mice per group were analyzed. Data are presented as mean ± SD (*n* = 5 random microscopic fields for each tumor). The treatment groups are indicated.

As further evidence of enhanced antitumor activity of Trast-NG/DOX or Trast-NG/DOX administered in combination with 17-AAG, we performed IHC analyses of tumors excised on day 2 after last injection. Treatment with Trast alone caused a significant reduction in proliferation (Ki67+ staining; *p* = 0.0033) but little increase in apoptosis (caspase-3+ cells) compared to the control group (5% Dextrose) (Figure 6 and Figure S5), consistent with a primarily cytostatic effect of Trast [40, 41]. Treatment with untargeted NG (IgG-NG/DOX) also reduced the percentage of Ki67+ cells (*p* = 0.0105) but had little impact on caspase-3+ cells (Figure 6 and Figure S5), consistent with a cytostatic mechanism of action of DOX [42, 43]. Notably, Trast-NG/DOX treatment led to a significant reduction in Ki67+ cells (*p* = 0.0023) as well as an increase in the percentage of caspase-3+ cells (*p* = 0.0059), and combined treatment with 17AAG further increased the impact of Trast-NG/DOX on both parameters (*p* = 0.0011 for Ki67+ cells and *p* = 0.0012 for caspase-3+ cells) (Figure 6), indicating that the combination is superior and promotes substantial cytotoxicity as compared to a primarily cytostatic effect of Doxorubicin [42, 44]. 17AAG also improved the pro-apoptotic efficacy of IgG-Trast/DOX but its impact on the anticancer activity of Trast-NG/DOX was substantially more pronounced, especially on the percentage of caspase-3+ cells in tumors (Figure 6 and Figure S5).

To mechanistically link the enhanced anti-tumor response of Trast-NG/DOX + 17-AAG combination with the effects of 17-AAG on ErbB2, we performed IHC and Western blot analyses of ErbB2 levels in tumors of mice treated with various regimens. Indeed, lower ErbB2 levels were seen in tumors of Trast-NG/DOX + 17-AAG treated mice (Figure S7), correlating with the superior antitumor efficacy of the combination.

Assessment of the impact of various treatment regimens on body weight indicated that the treatment-related toxicities associated with Trast-NG/DOX or Trast-NG/DOX + 17-AAG were generally mild (< 10% loss of body weight). In contrast, the same dose of DOX or the free drug combination (DOX + 17-AAG) produced a considerable body weight loss (18-20%, *p* < 0.05) (Figure S6). Light microscopic examination of H&E-stained tissue sections of liver, lung, kidney, spleen and heart resected from mice euthanized at the end of the experiment showed no histopathological changes in Trast-NG/DOX or Trast-NG/DOX + 17AAG treatment groups compared to the 5% Dextrose control group (data not shown), confirming the relatively low toxicities of Trast-based NGs.

## DISCUSSION

Introduction of targeted cancer nanomedicines into clinical practice has provided substantial momentum for the development of similar approaches against a variety of cancers. Most nanoparticle-sized therapeutic agents require internalization and traffic into acidic endocytic compartments to ensure the delivery of drug cargo into appropriate subcellular target [45, 46]. While much consideration has been given to the polymer design and the nature of encapsulated drugs to ensure successful delivery, the importance of the endocytic traffic routes of cell surface receptors that provide the basis for targeting of nano-carriers has received little attention. Here, we develop a proof-of-principle strategy, using ErbB2 as a targeting receptor, to help facilitate the entry of a model NG-encapsulated therapeutic agent Doxorubicin into lysosomes of breast cancer cells by tampering with the biological mechanism that retards the delivery of ErbB2 into lysosomes. Our studies demonstrate the feasibility of this novel approach in achieving superior *in vitro* targeting and *in vivo* anti-tumor efficacy in a breast cancer xenograft model. We suggest that this approach could significantly potentiate the efficacy of existing ErbB2-targeted nano-therapeutic agents against breast cancer. Furthermore, adaptation of this approach to facilitate lysosomal delivery of other receptors on cancer cells currently used or considered for targeting could substantially improve the efficacy of targeted cancer nanomedicines in general.

Our choice of ErbB2 receptor for proof-of-principle studies was based on several key considerations: ErbB2 is the driver oncogene for over 20% of breast cancers and smaller subsets of many other malignancies [47-49]; ErbB2 is the target of a clinically-approved prototype ADC, Trastuzumab-emtansine conjugate (Kadcyla^®^, Genentech) whose conjugated drug must be released to allow access to its cytosolic target [50]; ErbB2 is a prototype receptor that is biologically impaired in lysosomal entry [33, 35, 49]; studies by us and others have demonstrated that tampering with the function of a major biological partner of ErbB2 and a key to its inability to enter lysosomes, the molecular chaperone HSP90, promotes rapid lysosomal sorting and eventual degradation of ErbB2 [33, 35, 49]. ErbB2 is constitutively associated with HSP90, and multiple lines of investigation have demonstrated that HSP90 function is required for ErbB2 stability and ErbB2-driven oncogenesis [39, 51, 52]. Availability of specific HSP90 inhibitors has allowed a dissection of the mechanisms of ErbB2 instability upon HSP90 inhibition, and have revealed roles for lysosomal degradation [31-36]. Careful cell biological studies have revealed the lysosomal targeting of ErbB2 upon HSP90 inhibition to be a relatively early event, and one likely to be important for the rapid downregulation of ErbB2 from the surface of HSP90 inhibitor-treated cancer cells [31-36]. In contrast, lysosomal delivery of ErbB2 upon treatment of ErbB2+ cancer cell lines with Trast is distinctly slower and incomplete [32, 33], apparently due to the maintenance of a recycling itinerary of Trast-bound ErbB2 [53]. Our previous studies demonstrated that low concentrations of an HSP90 inhibitor, 17AAG, could however promote Trast-induced lysosomal delivery of ErbB2 and produce synergistic anti-tumor killing *in vitro* [32]. Indeed, Trast + 17AAG exhibit enhanced antitumor activity *in vivo* [32]. However, 17AAG and other HSP90 inhibitors have been slower to be adopted clinically as they produce significant toxicity [54-59]. The focus of such studies has been on the use of maximally-tolerated doses of HSP90 inhibitors, even when these are combined with other therapeutic agents [55-58, 60-64]. Our studies help establish a new paradigm in which HSP90 inhibitors at sub-therapeutic, and non-toxic, doses can substantially improve the efficacy of a nano-formulated therapeutic agent without significant added toxicity.

Our studies provide support for a potentially novel use of HSP90 inhibitors to facilitate the endolysosomal delivery of ADCs or antibody-conjugated drug nano-particles (ANPs) that rely on targeting of receptors that are HSP90 clients. A number of such targets have been identified, including the mutant EGFR in non-small cell lung cancer [65-68], a receptor we have shown to constitutively enter the endocytic recycling pathway [8, 68]. Similarly, glioblastoma-associated EGFR-vIII, which lacks the normal EGFR's ability to undergo ligand-triggered lysosomal traffic, is HSP90-associated [69]. Recent studies also suggest that overexpressed EGFR in gastric cancer cells is efficiently targeted by HSP90 inhibitors for degradation [70]. Thus, our approach using low doses of an HSP90 inhibitor is likely to be applicable to other malignancies as ADCs or ANPs against other HSP90-dependent receptors are developed for clinical use. While our studies relied on 17AAG as an HSP90 inhibitor, a number of other HSP90 inhibitors are being evaluated in clinical trials, including ones with oral bioavailability [57, 60, 61], and testing of such drugs in the context of ANPs and ADCs is highly desirable. Since HSP90 inhibition eventually promotes the degradation of targeted receptors, such as ErbB2 [32, 39, 71, 72], the scheduling of the ANP and HSP90 inhibitor is likely to be of key importance. In our studies, we considered this issue upfront and demonstrated the ability of HSP90 inhibition to facilitate the internalization and lysosomal targeting of Trast-NG *in vitro* (Figure 3). Accordingly, our preclinical therapy was designed to administer the HSP90 inhibitor 17AAG 2h hours after injecting nanogels, allowing time for the latter to reach their target. It remains possible that further studies to optimize the scheduling of HSP90 inhibitor relative to ANP will help improve the efficacy of regimens targeting ErbB2 or other HSP90 client receptors.

Our studies also provide evidence that sub-therapeutic doses of an HSP90 inhibitor (1 mg/Kg 17AAG in our studies, which has little efficacy against BT-474 xenografts; unpublished data), dramatically enhances tumor killing by DOX-loaded ErbB2-targeted NGs (Figure 5A). Such a combination led to not just inhibition of xenograft tumor growth but in fact a significant shrinkage of tumor masses from their pre-treatment volume (Figure 5A). This was associated with a significant shift from a predominantly cytostatic effect, as expected [42-44], to a significantly cytotoxic effect on tumors as demonstrated by Ki67 and caspase-3 staining of tumors among surviving mice at the end of therapy. Not surprisingly, combination with low dose 17AAG markedly improved the survival of tumor-bearing mice (Figure 5B). We must point out that DOX was used in our proof-of-principle studies as the polymer design we have previously established allows efficient loading of this clinically-used chemotherapeutic agent [37]. However, DOX enhances the cardiotoxicity associated with ErbB2-targeted antibody therapeutics, including Trastuzumab [73-75], and as such the approach will need modifications to incorporate drugs more appropriate for ErbB2+ cancer therapy. Such studies are currently ongoing in our laboratories. The current nano-carrier design should however be applicable to other tumors where a targeting antibody or other moiety is available.

Based on our studies, we suggest that the endocytic traffic itinerary of receptors selected for targeting of ADCs and ANPs should receive careful consideration. An understanding of mechanisms that may impair the endolysosomal traffic of a targeted receptor (as with ErbB2) may help overcome an efficacy barrier for newer agents in development and potentially improve the efficacy of agents already in clinic. In this regard, the potential combination of HSP90 inhibitors that are currently in clinical trials together with ErbB2-targeted TDM-1 will be of considerable interest. A recently FDA-approved ADC, Brentuximab-Vedotin, targets CD30 receptor in Hodgkin's lymphomas [76, 77] which requires lysosomal traffic for its efficacy [5], although the mechanisms of lysosomal trafficking of CD30-bound ADC remain unknown.

It is of interest that HSP90 is also thought to regulate the activity of Rab GTPase-dependent endocytic recycling by its association with a Rab Guanine Nucleotide Dissociation Inhibitor (GDI) [78, 79], and HSP90 inhibitors could potentially help attenuate Rab-dependent recycling of transmembrane receptors that are not direct HSP90 clients to enhance the intracellular delivery of ADCs or ANPs. Other factors that may control trafficking of oncogenic cell-surface receptors such as the non-receptor tyrosine kinase c-Src, endocytic adaptor proteins, E3 ubiquitin ligases, deubiquitinating enzymes, RAB GTPases, ESCRT proteins and EHD family proteins, [80-83] may offer future opportunities to develop facilitators of ADC and ANP traffic to endolysosome compartments as approaches of therapeutic potentiation.

## CONCLUSIONS

Our study demonstrates the potential of therapeutically exploiting the vulnerabilities in cancer cell-specific trafficking behavior of receptors used to selectively deliver nano-encapsulated therapeutic agents, and highlights the importance of identifying such vulnerabilities in target receptors in order to fully realize the potential of ADCs and targeted nanomedicines in cancer and other diseases.

## MATERIALS AND METHODS

### Materials

Trastuzumab was obtained from UNMC Pharmacy. PEG-*b*-PMA di-block copolymer (Mw/Mn = 1.45) was purchased from Polymer Source Inc., Canada. The block lengths were 170 and 180 repeating units for PEG and PMA, respectively. The concentration of carboxylate groups in the copolymer samples was estimated by potentiometric titration. FMOC-PEG-NHS was purchased from Creative PEGWorks, NC. Calcium chloride, Ethylenediamine, 1-(3-dimethylaminopropyl)-3-ethylcarbodiimide hydrochloride (EDC) and Ethylenediaminetetraacetic acid (EDTA) were obtained from Sigma-Aldrich (St Louis, MO). Doxorubicin hydrochloride was kindly provided by Dong-A Pharmaceutical Company, South Korea. Fetal bovine serum (FBS) (dialyzed and heat-inactivated), Dulbecco's Modified Eagle's Medium (DMEM), RPMI-1640 medium and Lysotracker™ (green) were purchased from Invitrogen Inc. (Carlsbad, CA). MTT reagent (3-(4,5-Dimethylthiazol-2-yl)-2,5-diphenyltetrazolium bromide) was purchased from Research Products International (Prospect, IL). All other chemicals were of reagent grade and used without further purification. The following primary antibodies were used in this study: mouse monoclonal anti-human-ErbB2 (raised against the C-terminal 1242-1255 amino acid residues) used for Western blotting studies from BD Pharmingen™ (San Diego, CA); goat anti-human-ErbB2 polyclonal (AF1129) used for flow cytometry and immunofluorescence studies from R&D Systems (Minneapolis, MN); mouse monoclonal anti-phosphotyrosine (anti-pY; 4G10) kindly provided by Dr. Brian Druker (Oregon Health & Science University, Portland, OR); and mouse monoclonal anti-Hsc70 from Santa Cruz Biotechnology Inc. (Santa Cruz, CA).

### Synthesis of the NGs

NGs were synthesized essentially as described [37, 38]. Briefly, PEG-*b*-PMA/Ca^2+^ complexes were prepared by mixing an aqueous solution of PEG-*b*-PMA with a solution of CaCl_2_ at a molar ratio of [Ca^2+^]/[COO−] = 1.3. The chains were cross-linked overnight at room temperature (RT, using EDC and 1,2-ethylenediamine ([EDC]/[1,2-ethylenediamine] = 2; [COOH]/[EDC] = 5). The synthesized NGs were extensively dialyzed (MW Cut-off 3.5 kDa) against 0.5% aqueous ammonia in the presence of 100 mM EDTA followed by double distilled water.

### Synthesis and characterization of Trastuzumab-modified and DOX-loaded NGs

Trastuzumab (Trast) or mouse IgG (control) conjugation to NGs through a streptavidin-biotin complex (Figure 1) followed the following steps:

#### PEGylation of streptavidin

6 ml of 1 mg/ml (16.7 μM) streptavidin in 50 mM sodium bicarbonate buffer (pH 8.3) was added dropwise to 1 ml of 1.5 mg/ml (150 μM) solution of Fmoc-PEG-NHS (Mw = 10 kDa; 1.5 fold molar excess with respect to streptavidin) in the same buffer. The mixture was incubated for 2h at RT. Fmoc groups were removed by adding 0.5 μL of 20% piperidine/DMF (5 fold molar excess over Fmoc) and incubating the reaction mixture for 10 min at RT. The resulting construct (NH_2_-PEG-streptavidin) was purified through repeated ultrafiltration (MW Cut-Off 30,000, Millipore) at 3000 rpm for 15 min (3 washes with water) to remove unreacted PEG. The absence of free PEG was confirmed by size exclusion chromatography (SEC) on a Sepharose CL-6B-based column, using an ÄKTA FPLC (Amersham Biosciences).

#### Insertion of NH2-PEG-streptavidin into NGs

Carboxylate groups within the NG core were activated with 300 μL of 1 mg/ml EDC solution for 15 min. A 10-fold excess of EDC with respect to NH2-PEG-streptavidin (to be added in the subsequent step) was used to ensure maximal incorporation of NH_2_-PEG-streptavidin into the NG core. Insertion of NH_2_-PEG-streptavidin into NGs was achieved through the reaction between free NH_2_ groups of PEG with EDC-activated carboxyl acid core of NGs. The mixture was allowed to react for 2 h followed by purification of streptavidin-NG conjugate using repeated ultrafiltration (MW Cut-Off 100,000, Millipore) at 4500 rpm for 30 min (3 washes with water) to remove any unreacted PEG-Streptavidin. The absence of free PEG-Streptavidin was confirmed by SEC, as above. The amount of streptavidin n the streptavidin-NG conjugate was measured by the Bradford method using a BioRad assay kit (BioRad, Richmond, CA, USA) with BSA as standard.

#### Preparation of Trast-NG *via* streptavidin-biotin complex, and drug loading

Trastuzumab (or control IgG) was biotinylated using EZ-Link™ Sulfo-NHS-Biotin (ThermoFisher Scientific) according to the manufacturer's protocol. Streptavidin-NGs were mixed with biotinylated Trastuzumab (or control IgG) at a 1:2 streptavidin to biotin ratio and incubated for 2h at RT. The mAb-NG was purified by SEC, to remove all unbound mAb, yielding a mixture of unmodified NG and mAb-NGs. The amount of Trast or IgG in conjugated NGs was measured by the Bradford method. Streptavidin-NG alone was used as a control to subtract the values contributed by Streptavidin portion to the total protein in the mAb-NG. DOX-loaded Trast-NGs or control IgG/NGs were prepared by adding DOX to an aqueous dispersion of NGs (pH 7.0) at a feeding ratio (R) of [Dox]/[COO^−^] = 0.25 and the mixture incubated for 24 h. Unbound DOX was removed by repeated ultrafiltration (MW Cut-Off 30,000, Millipore), as above.

### Determination of NG particle size and zeta-potential

The intensity-mean Z-averaged particle diameter (D_eff_) and ζ-potential of NGs were measured at 25°C using a Malvern Zetasizer (Malvern Instruments Ltd., Malvern, UK), set in an automatic mode. The vendor-provided software was used to calculate size, polydispersity indices and ζ-potential of NGs from measurements performed at least in triplicate.

### Cell culture conditions

The ErbB2-overexpressing breast cancer cell lines SKBr-3, 21MT1 and BT-474, and a representative ErbB2-low line MCF-7 were maintained, as previously described [32, 84]. The cell lines were obtained from ATCC, except for 21MT-1, which has been described by one of the coauthors, Dr. Vimla Band [85].

### Confocal microscopy

To examine the cellular uptake and subcellular localization of Trast-NG by confocal microscopy (Carl Zeiss LSM 510 Meta, Peabody, MA), SKBr-3 cells were plated on glass slides placed within 12-well plates (1×10^5^ per well). Trast-NGs without DOX were incubated with SKBr-3 cells at 37°C for 1h. 17-AAG (100 nM) was then added for 2, 4 or 8h, and cells washed in PBS and fixed in 4% parafolmaldehyde in PBS. Trast-NGs were visualized by staining Trastuzumab with FITC-conjugated mouse anti-Human monoclonal antibody (green). ErbB2 was stained either with a goat anti-ErbB2 antibody (AF1129) against the extracellular domain of ErbB2 or a mouse monoclonal antibody against the C-terminal tail of ErbB2 (BD Pharmingen) followed by Alexa594-conjugated anti-goat or anti-mouse antibody, respectively. Lysosomes were identified by staining with mouse anti-human LAMP-1 monoclonal antibody followed by Alexa-594-conjugated goat anti-mouse antibody.

### Cell cycle analysis

To assess the effect of DOX to arrest the cell cycle [84], 21MT-1 or MCF-7 cells were seeded in 6-well plates for 24h and exposed to various concentrations of Trast-NG/DOX (1.7 - 13.3 μM Dox) for 6h, followed by a further 18h incubation without (control) or with 100 nM 17-AAG. Cells were stained with propidium iodide (PI) and cell cycle distribution assessed by FACS analysis [32, 84].

### Analysis of NGs against BT-474 tumor xenografts

10^7^ BT-474 cells in 100 μL of 50% Matrigel (BD Biosciences, California) in media were injected into mammary fat pads of 4-6 week old female nude mice (Athymic NCr-nu/nu; NCI Fredrick National Lab, Fredrick, MD) that had received subcutaneous 17-β-Estradiol pellets (0.72mg/pellet; 60 day release; Innovative Research of America, Sarasota, FL) on the lateral side of the neck 3 days earlier. 14 days later, mice with tumor volumes ranging between 100-200 mm^3^ were stratified into groups and started on the following treatments *via* tail vein injections every 4 days: 1) 5 % dextrose (Control); 2) Trast alone (3 mg/kg); 3) IgG-NG/DOX (6 mg/kg); 4) IgG-NG/DOX (6 mg/kg) + free 17AAG (1 mg/kg); 5) Trast-NG/DOX (6 mg/kg); 7) Trast-NG/DOX (6 mg/kg) + free 17AAG (1 mg/kg). 17-AAG was administered 2 h after NGs. Body weight and tumor volume were monitored every other day. Tumor volume (V = 0.5 x L x W^2^) was estimated by measuring two orthogonal diameters (longer dimension: L, and smaller dimension: W) of the tumor using electronic calipers. Mice were sacrificed when tumor volume exceeded 2000 mm^3^, greatest tumor dimension exceeded 20 mm, tumor became necrotic, or animals lost more than 20% of body weight. All other animals were sacrificed by day 78 after start of treatment. All animal studies were approved by the Institutional Animal Care and Use Committee (IACUC).

### Apoptosis and proliferation

Tumors were excised from 2-3 tumor-bearing mice per treatment group treatments at day 14 of treatment, fixed in 10% neutral buffered formalin, paraffin embedded (UNMC Tissue Sciences Facility, Omaha, NE). 4-μm thick tissue sections were analyzed for proliferating and apoptotic cells using immunohistochemistry (IHC) with antibodies against Ki-67 and caspase-3, respectively, with visualization with DAB+ (brown for Ki-67; permanent red for caspase-3) (DAKO) for 2 min. The sections were rinsed with distilled water and counterstained with hematoxylin. Ki-67 and caspase-3 expression was quantified by determining the area occupied by positively-stained cells (using ImageJ software) in 5 random high-power fields (at 20× magnification) and dividing by the total area of cells per field.

### Histopathology analysis

Liver, spleen, heart, kidney and lungs were excised and fixed in buffered formalin and paraffin-embedded. Serial 5-μm sections were stained with hematoxylin and eosin (H&E) and histopathological analyses carried out by a pathologist (SMC) under a light microscope.

### Statistical analysis

Differences between treatment groups were analyzed for significance using the Student *t*-test assuming a type I error (α) = 0.05. For *in vivo* antitumor effects and toxicity analyses, group means for tumor volume and body weights, respectively, were evaluated using the repeated measures analysis of variance. Survival was estimated using Kaplan-Meier analysis and compared using the log-rank test. *P* values < 0.05 were considered significant. Analysis of variance and Kaplan-Meier analysis were performed using GraphPad Prism 5 (GraphPad Software, Inc.). Median survival time for the various groups and the associated Confidence Interval limits were derived by running PROC LIFETEST in SAS (Statistical Analysis System) software.

## SUPPLEMENTARY MATERIAL FIGURES AND TABLES


