# Supplementary material for: Marked enhancement of lysosomal targeting and efficacy of ErbB2-targeted drug delivery by HSP90 inhibition
Source: Oncotarget. 2016 Feb 7;7(9):10522–35. doi: 10.18632/oncotarget.7231 (PMC4891137; doi:10.18632/oncotarget.7231)
Supplement: Supplementary file 1 [file oncotarget-07-10522-s001.pdf]

Marked enhancement of lysosomal targeting and efficacy of ErbB2-targeted drug delivery by HSP90 inhibition

Supplementary Material

1.1. Purification of Trast-NG

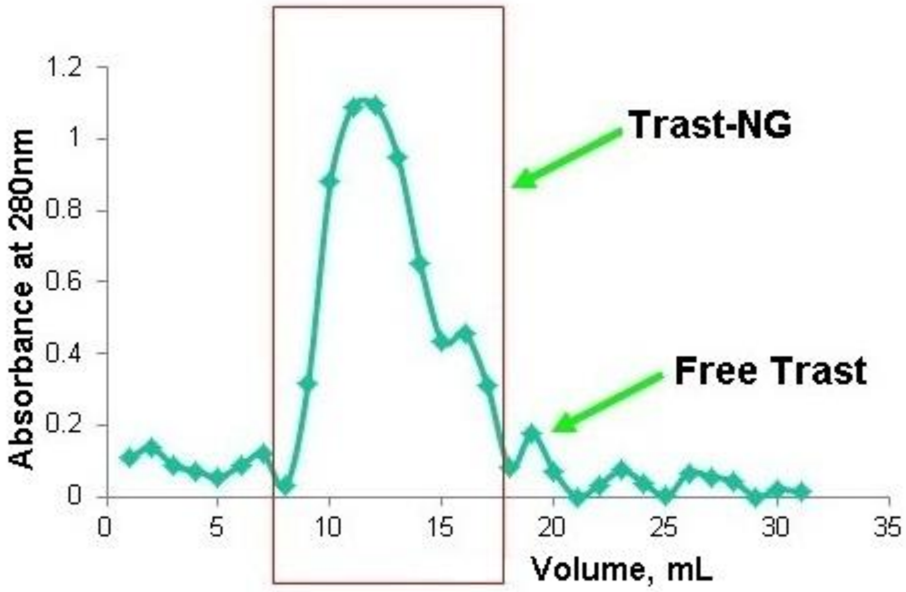

Fig. S1. Purification of Trast-NG by FPLC

1.2. Colocalization of ErbB2 and Trast-NG

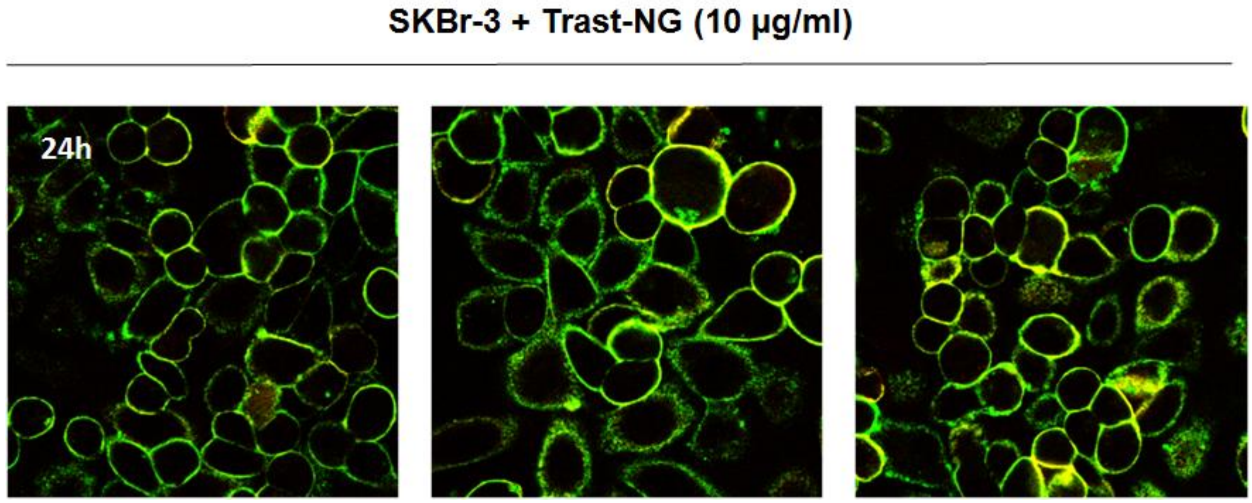

Fig. S2. Binding of Trast-NG to ErbB2-overexpressing breast cancer cells was confirmed by confocal microscopy. ErbB2-was visualized using a mouse monoclonal antibody directed against the C-terminal antigenic region of ErbB2 (BD Pharmingen), followed by Alexa594-conjugated anti-mouse secondary antibody. The Trast-NG bound to the ErbB2 was detected using a FITC-conjugated goat anti-human secondary antibody to detect the Fc portion of the Trast-conjugated to the NG.

1.3. In vitro cytotoxicity study.

Cytotoxicity of drug-loaded NGs was assessed in BT-474 and MCF-7 cells by a standard MTT assay (1). Briefly, cells were seeded in 96-well plates (5,000 cells/well) and allowed to adhere for 24 h prior to the assay. Cells were exposed to various concentrations of Trast-NG/DOX and NG/DOX (0 - 10 µg/mL Dox) for 48 h at 37 °C. Cells were then washed with PBS and MTT indicator dye (25 µL, 5 mg/mL) was added to each well and the cells were incubated for 2 h at 37°C in the dark. A solution of 50% DMF-20% SDS (100 µL) was added to each well and kept overnight at 37 °C. Absorbance was read at 570 nm using a plate reader (SpectraMax M5, Molecular Devices Co., USA). Cell survival rates were calculated as normalized to control untreated wells. All measurements were taken eight times. Based on the results of the test, the IC_{50} values were calculated by using GraphPad Prism 5 (GraphPad Software, Inc.).

Table S1 . Comparison of IC_{50} values for targeted Vs non-targeted DOX-loaded nanogels ErbB2 high and ErbB2 low cells as determined by the MTT assay.

	IC_{50} (μM)	
	BT-474 (ErbB2 +)	MCF-7 (ErbB2 -)
NG/DOX	0.43 ± 0.13	0.29 ± 0.1
Trast-NG/DOX	0.07 ± 0.08	0.19 ± 0.07

1.4. Kinetics of endocytic uptake of Trast-NG into ErbB2-overexpressing breas cancer cells

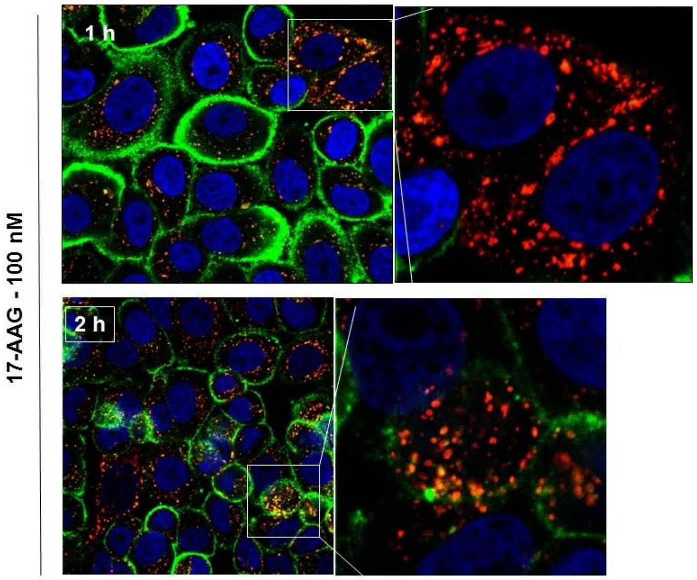

Fig. S3. SKBR-3 cells grown on glass coverslips were treated with Trast-NG followed by 17-AAG as described in the legend for Fig 3 A. The internalized Trast-NG (seen in green) was visualized as described in the legend for Fig 2. The lysosomal marker LAMP-1 (seen in red) was stained using mouse anti-human LAMP-1 mAb, followed by Alexa-594-conjugated anti-mouse secondary. Co-localization of Trast-NG with LAMP-1 can be seen as regions in yellow/orange.

1.5. *In vivo* antitumor efficacy of Trast-NG/DOX in ErbB2-low cancer xenograft-bearing female nude mice

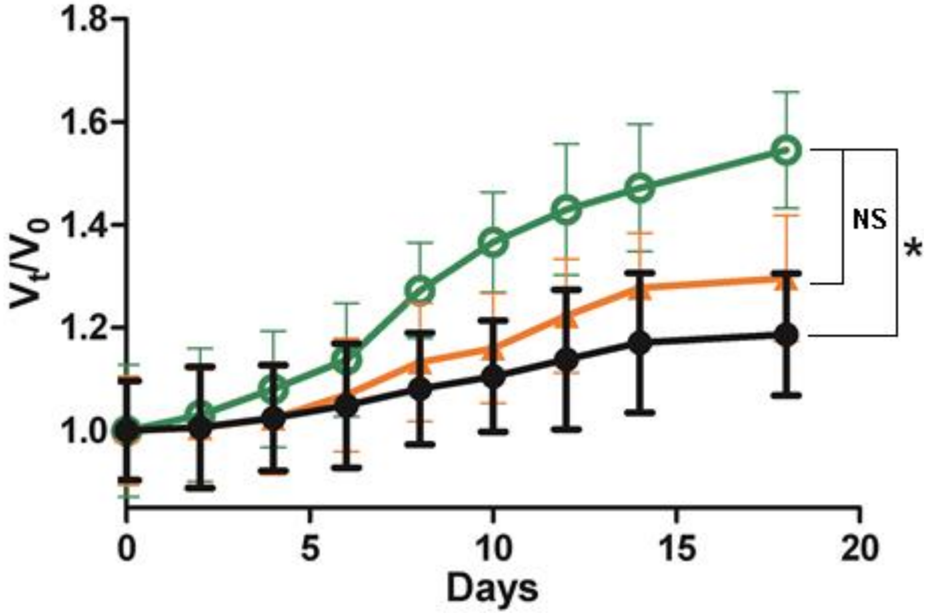

Fig. S4. Relative changes in tumor volume after administration of ErbB2-targeted Trast-NG/DOX in ErbB2 - breast cancer mice model. Trast-NG/DOX (●) or IgG-NG/DOX (▲) or Control (Ø).

1.6. Ki-67-caspase-3 apoptosis assay

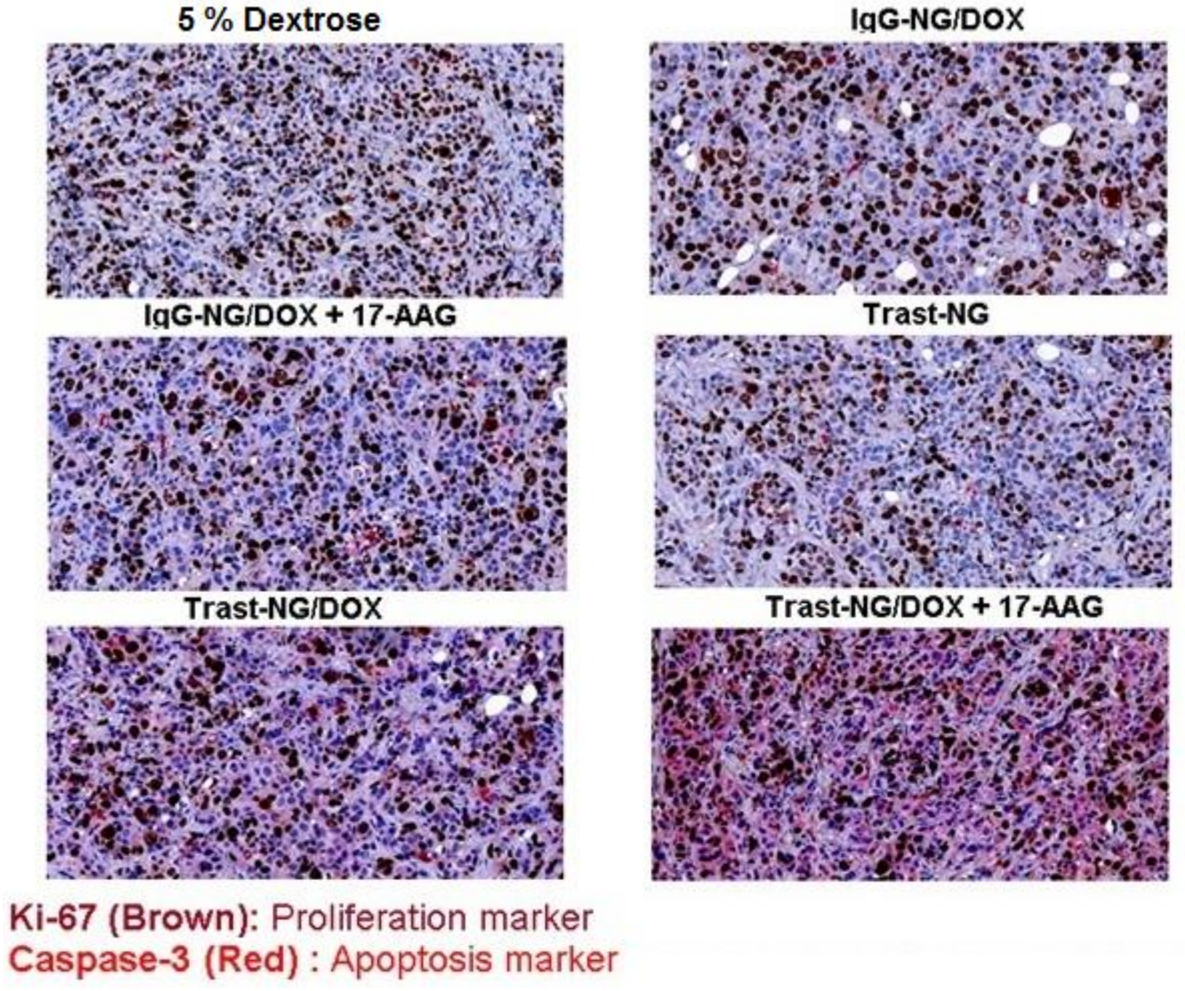

Fig. S5. Ki-67-caspase-3 apoptosis assay.

1.7. Changes in the body weight of mice following treatments

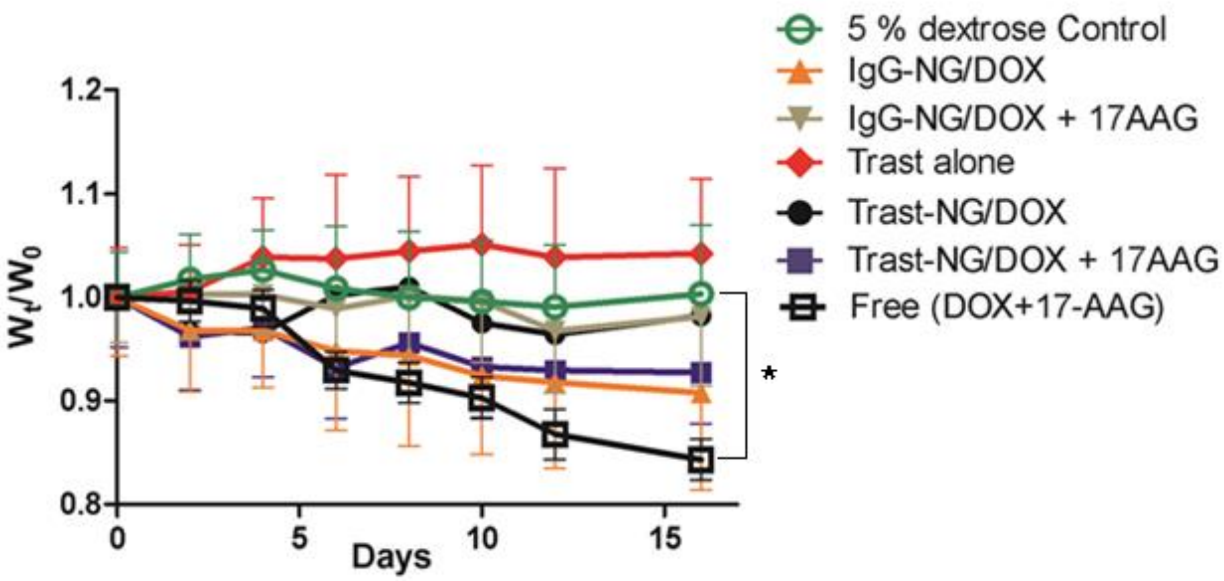

Fig. S6. Changes in body weight are presented as a fold-ratio compared baseline for each animal on day 0 of treatment. Values represent mean ± SEM.

1.8. ErbB2 degradation in tumor sections

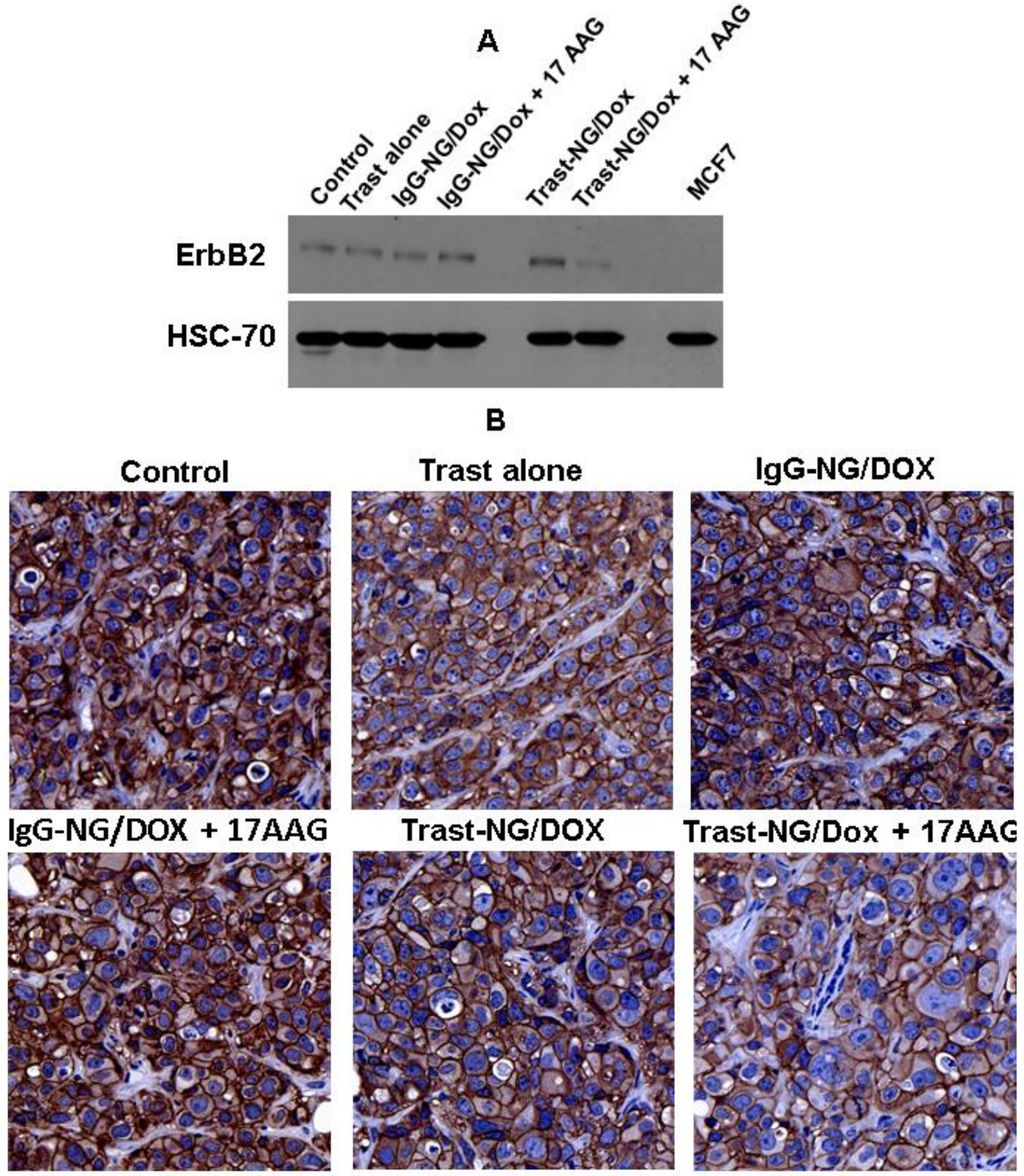

Figure S7. ErbB2 levels analyzed in the tumor sections using A) western blot and B) ErbB2-IHCs.

Table S2: Comparison of Median survival (in days), the associated Confidence Intervals and the p-values (with respect to 5% Dextrose control) are tabulated in Table S2

Treatment Group	Median Survival (days)	Confidence Interval (days) (Lower limit – Upper limit)	p-values (vs. 5% Dextrose control	% Survival at censoring date (76 days)
**Saline**	40	26 - NE		0
**Trast**	48	36 - NE	0.1794	0
**IgG-NG/Dox**	52	26 - NE	0.3221	0
**IgG-NG/Dox + 17-AAG**	40	32 - NE	0.2827	0
**Trast-NG/Dox**	NE	68 - NE	0.0032	56.8
**Trast-NG/Dox + 17-AAG**	NE	NE - NE	<0.0001	88.8

NE - Estimate not reached

References

1. Ferrari M, Fornasiero MC, Isetta AM. MTT colorimetric assay for testing macrophage cytotoxic activity in vitro. J Immunol Methods 1990;131(2):165-72.
